# Oxidative Stress Links Aging-Associated Cardiovascular Diseases and Prostatic Diseases

**DOI:** 10.1155/2021/5896136

**Published:** 2021-07-17

**Authors:** Ming-Juan Zhao, Shuai Yuan, Hao Zi, Jia-Min Gu, Cheng Fang, Xian-Tao Zeng

**Affiliations:** ^1^Center for Evidence-Based and Translational Medicine, Zhongnan Hospital of Wuhan University, Hubei, Wuhan 430071, China; ^2^Department of Urology, Zhongnan Hospital of Wuhan University, Hubei, Wuhan 430071, China

## Abstract

The incidence of chronic aging-associated diseases, especially cardiovascular and prostatic diseases, is increasing with the aging of society. Evidence indicates that cardiovascular diseases usually coexist with prostatic diseases or increase its risk, while the pathological mechanisms of these diseases are unknown. Oxidative stress plays an important role in the development of both cardiovascular and prostatic diseases. The levels of oxidative stress biomarkers are higher in patients with cardiovascular diseases, and these also contribute to the development of prostatic diseases, suggesting cardiovascular diseases may increase the risk of prostatic diseases via oxidative stress. This review summarizes the role of oxidative stress in cardiovascular and prostatic diseases and also focuses on the main shared pathways underlying these diseases, in order to provide potential prevention and treatment targets.

## 1. Introduction

Cardiovascular diseases (CVDs), including hypertension, coronary heart disease (CHD), cerebrovascular disease, and heart failure, are the major cause of death globally. In the period from 1990 to 2019, the prevalence of total CVD nearly doubled from 271 million to 523 million cases, and the number of CVD deaths steadily increased from 12.1 million to 18.6 million [1]. In 2019, ischaemic heart disease was one of the top-ranked causes of disability adjusted life years (DALYs) in both the 50-74-year and 75-years-and-older age groups [2]. According to World Health Organization (WHO) statistics, almost 23.6 million people will die from CVDs by 2030. Benign prostatic hyperplasia (BPH) and prostate cancer are also aging-associated diseases. The incidence rate of BPH increases with age affecting about 50% of men over 50 years, increasing to 80% when they reach 80 or above [3, 4]. There is also an increased incidence in prostate cancer cases from 940,000 in 2007 to 1.3 million in 2017 [5], and the age-standardized incidence of prostate cancer in China also rose by 2.75% from 1990 to 2017 [6]. Aging clearly plays an important role in CVDs (such as hypertension and CHD) and prostatic diseases (prostate cancer and BPH).

The current evidence suggests that CVDs usually coexist with prostatic diseases or increase its risk, and there are 13 related clinical studies showing CVD as a risk factor for prostatic diseases (see Table 1); however, whether there is a causal relationship between them is still controversial. Numerous studies have reported that oxidative stress can promote the occurrence and development of both prostatic diseases and CVDs [7–10]; hence, to summarize its role in these two diseases can provide information for seeking prevention and potential therapeutic targets.

## 2. Oxidative Stress

**Figure 1:**
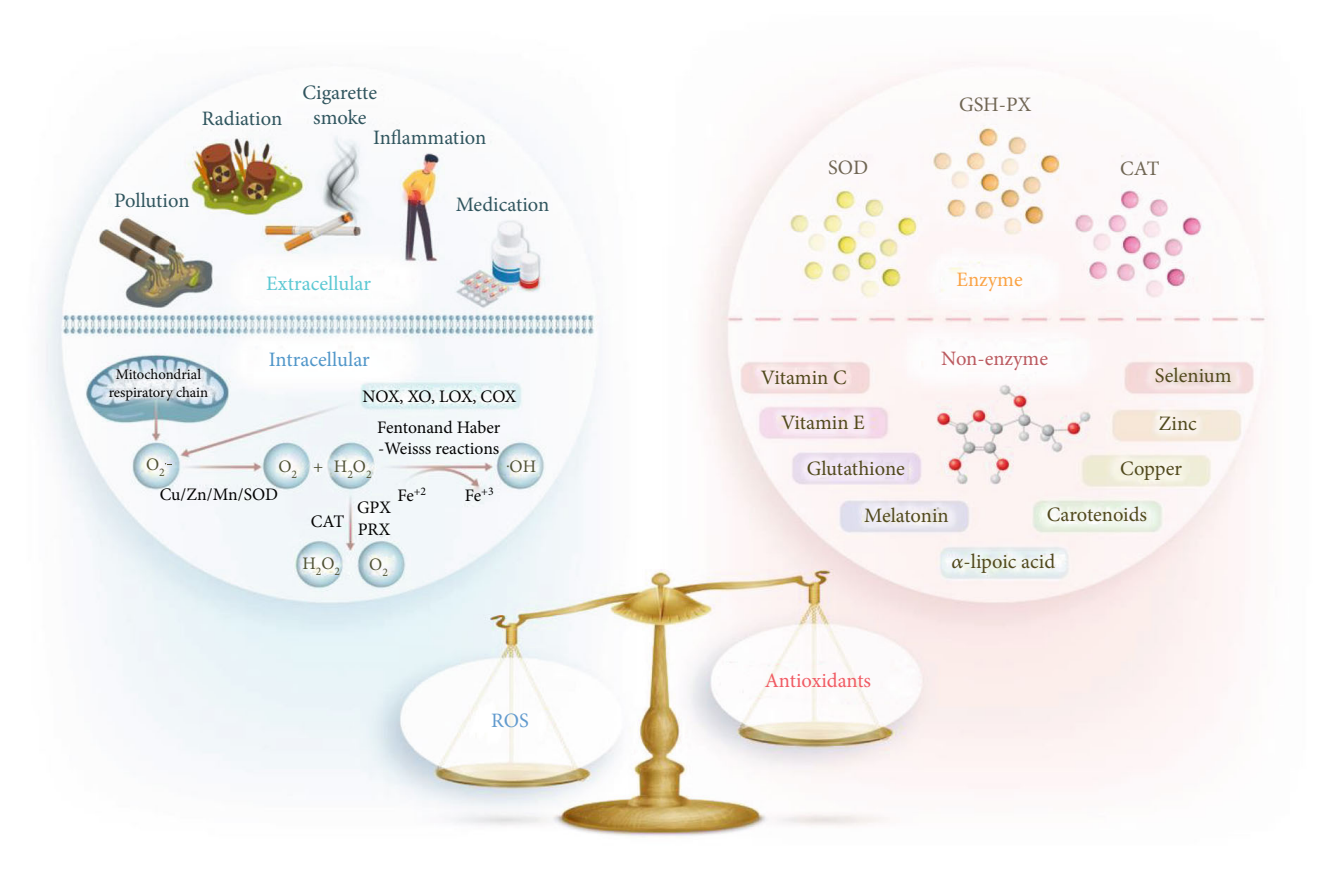
Schematic representation of the oxidative stress theory and the sources of ROS and antioxidants. When the generation of ROS outweighs antioxidative capacity, this leads to oxidative stress. ROS are generated from extracellular and intracellular sources. The extracellular sources of ROS are pollution, inflammation, cigarette smoke, radiation, and medication. Intracellular sources of ROS are the mitochondrial electrotransport chain: NADPH oxidase (NOX), xanthine oxidase (XO), lipoxygenase (LOX), and cyclooxygenase (COX). The enzyme antioxidant system includes superoxide dismutase (SOD), catalase (CAT), glutathione peroxidase (GSH-PX), and the nonenzyme antioxidant system includes vitamin C, vitamin E, glutathione, melatonin, *α*-lipoic acid, carotenoids, and trace elements such as copper, zinc, and selenium.

The concept of oxidative stress originated from human understanding of aging. In 1956, Professor Harman first proposed the theory of free radical aging. In 1990, Professor Sohal pointed out the flaws of this theory and put forward the concept of oxidative stress for the first time [24, 25]. Oxidative stress is a situation where the balance of oxidative systems and antioxidative systems *in vivo* is changed in favour of the former [26, 27]. Oxidants are regulators of normal cellular function, but when the production of reactive oxygen species (ROS) exceeds the scavenging capacity, the oxidation and antioxidant systems will be unbalanced, causing damage to the tissues and cells; this phenomenon is called oxidative damage [28–30]. ROS includes superoxide anion (O2^•-^), hydroxyl radical (•OH), and hydrogen peroxide (H_2_O_2_). To maintain the balance, there are two antioxidative systems in vivo: one is the enzyme antioxidant system, including superoxide dismutase (SOD), catalase (CAT), and glutathione peroxidase (GSH-PX), and the other is the nonenzyme antioxidant system which includes vitamin C, vitamin E, glutathione, melatonin, *α*-lipoic acid, carotenoids, and trace elements such as copper, zinc, and selenium (SE).  Figure 1 presents the oxidative stress theory and the sources of ROS and antioxidants.

### 2.1. Characteristics of ROS

ROS are generated by a variety of extracellular and intracellular actions (Figure 1). The main intracellular source of ROS is the mitochondrial respiratory chain. The O2^•-^ is the principal ROS formation which is produced by the enzymatic reaction and a nonenzymatic electron transfer reaction in cell. The enzymes that generate the superoxide include NADPH oxidase (NOX), xanthine oxidase (XO), lipoxygenase (LOX), and cyclooxygenase (COX) [31]. H_2_O_2_ is produced from O2^•-^ by enzymatic dismutation by the three isoforms of Cu/Zn/Mn SOD in intracellular. Most of the H_2_O_2_ is converted to H_2_O by the catalase (CAT), glutathione peroxidase (GPX), and peroxiredoxins (PRX). H_2_O_2_ can damage DNA when it is converted to a •OH through Fenton and Haber-Weiss reactions in the transition metal ions, especially iron ions (Fe^+2^). The •OH is the most toxic form of ROS which causes various types of DNA damage, lipid peroxidation, and protein modification [32]. The extracellular sources of ROS commonly included pollution, inflammation, cigarette smoke, radiation, and medication [33].

At present, accurately measuring ROS in disease is still a problem. ROS are unstable, and their half-lives are relatively short. For example, the half-life of O2^•-^ is 10^−6^ to 10^−9 seconds^, and the •OH is 10^−9 seconds^ [34, 35]. Therefore, ROS are routinely measured by biomarkers of oxidative damage, which included markers of protein damage (protein carbonyl derivatives), lipid peroxidation (malondialdehyde [MDA] and 4-hydroxynonenal [HNE]) [36], and DNA oxidation (8-hydroxy-2′-deoxyguanosine [8-OHdG]). Although, the biomarkers of measured oxidative stress are indirect and have low specificity, these do provide a noninvasive method in clinical practice.

## 3. Pathophysiological Role of Oxidative Stress in CVD and Prostatic Diseases

**Figure 2:**
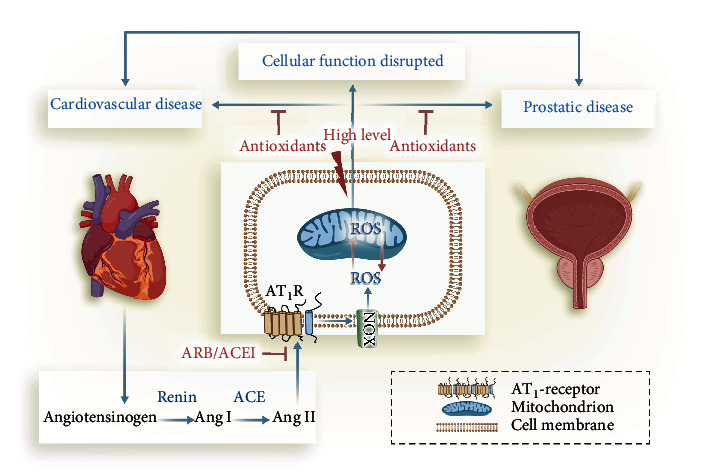
ROS generated by NOX in the pathogenesis between CVD and prostatic diseases. The renin-angiotensin system (RAS) exists in both heart and prostate; in addition, the overactivity of the RAS is found in both cardiovascular diseases (CVD) and prostatic diseases. The RAS includes angiotensinogen, renin, angiotensin conversion enzyme (ACE), angiotensin II (Ang II), and angiotensin receptors. Ang II is a biologically active peptide in RAS, and its main effector receptor is the type 1 (AT_1_R). Ang II induced the ROS by activation of the subunits of NADPH oxidase (NOX), and then, the increased ROS effects the development of CVD and prostatic diseases. Thus, NOX-derived ROS signal may be a common potential target in therapeutic intervention of CVD and prostatic diseases.

Numerous clinical studies have indicated that oxidative stress plays a role in CVD and prostatic diseases (Table 2). Studies have reported that the level of oxidative stress biomarkers was higher in CVD patients [37–40]. Additionally, current evidence suggests that oxidative stress is associated with the etiology and pathogenesis of the prostatic diseases [9, 41–43]. Hence, the phenomenon of CVD increasing the risk of prostatic diseases may be attributed to oxidative stress (Figure 2).

### 3.1. Renin-Angiotensin System in CVD and Prostatic Diseases

It is well established that the activation of renin-angiotensin system (RAS) is critically associated with the pathogenesis of hypertension and atherosclerosis [58–60]. Previous studies have reported the existence of local RAS in the prostate, and the overactivity of the RAS may be involved in the pathophysiology of BPH and prostate cancer [61–64]. The RAS includes angiotensinogen, renin, angiotensin conversion enzyme (ACE), angiotensin II (Ang II), and angiotensin receptors. Ang II is a biologically active peptide in the RAS, and its main effector receptor is type 1 receptor (AT_1_R) (Figure 2).

The ACE inhibitors (ACEIs) and angiotensin receptor blockers (ARBs) have been successfully used as antihypertensive medication, and some studies have reported that these drugs have also been used in anticancer therapy [65, 66]. A meta-analysis which included 9 cohort studies with 20267 patients suggested that the use of ACEIs/ARB may be associated with a decreased risk of prostate cancer [65]. However, another meta-analysis of observational studies did not find a significant relationship between the use of ACEIs/ARB and prostate cancer risk [67]. Although it is still controversial, ACEIs or ARBs have been used to reduce the risk of prostate cancer. One study showed that the expression level of AT_1_R mRNA was higher in prostate cancer than that in the normal human prostate, based on the data, and ACEIs or ARB may inhibit prostate cancer [68]. Another study reported that the ACEI captopril lowers the risk of prostate cancer, but it was not significant [69]. In BPH patients, a clinical study showed that the use of ARB can improve prostatic hyperplasia [70], whereas other antihypertensive drugs were not effective, which indicates that ARB could ameliorate BPH independently of decreasing blood pressure. The ARB drug Losartan could treat the BPH in spontaneously hypertensive rats (SHRs). The study showed that long-term Losartan treatment restored prostatic blood flow and reduced tissue MDA (oxidative stress marker) in SHRs [71]. These findings suggest that ARB/ACEI may also effective in prostatic diseases.

Traditionally, it has been thought that Ang II can directly achieve vasoconstriction by interacting with AT_1_R in vascular smooth muscle. Recently, a novel signalling mechanism for Ang II-induced vascular superoxide (O_2_^−^) formation was associated with the development of endothelial dysfunction, hypertension, and atherosclerosis. The source of this increased O_2_^−^ seemed to be membrane-bound vascular NADPH oxidases (NOXs) [72, 73]. A study has also reported that angiotensin II also induced the production of ROS prostate cancer cells by upregulating the subunits of NADPH oxidase (NOX) [74]. In addition, another study has found expressions of various NOX isoforms in prostate cancer cell lines and a cross-talk between the endogenous ROS generation by NOX system and the tumorigenic potential [41]. Taken together, the ROS generated by NOX may play the central role connecting CVD and prostatic diseases.

### 3.2. NOX in CVD and Prostatic Diseases

NOXs are the key enzymes of redox signalling and also the main source of ROS in vivo [75]. ROS are generated by NOX in the pathogenesis of CVD (see Figure 2). In human cells, there are seven isoforms of the NOX family proteins, which are NOX1, NOX2, NOX3, NOX4, NOX5, DUOX1, and DUOX2. NOX1, NOX2, and NOX5 directly generate superoxide anions. Conversely, the NOX4 produces H_2_O_2_ which may be associated with its localization in the mitochondria in cardiomyocytes and in the endoplasmic reticulum in endothelial cells [76–78]. Superoxide cannot cross the membranes of these organelles. DUOX1 and DUOX2 also produce H_2_O_2_ [79]. The transmembrane subunit P22^phox^ and NOX subtypes (NOX1, NOX2, and NOX4) make up the membrane-bound catalytic core [80]. The NOX4 activity requires P22^phox^ and is also regulated by POLDIP2. Of the seven NOXs, NOX1 and NOX2 play a role in immune defence [81], other NOXs act as the second messenger to participate in the regulation of cell signal pathways and maintain the stability of the intracellular environment [82].

#### 3.2.1. NOX and CVD

The association between increased vascular ROS production and hypertension has been reported in animal models with Ang II induced via NOX activation [72]. Some recent in vitro studies have suggested a pivotal role of the NOX and its subunit p47phox in vascular oxidant stress and the blood pressure response to angiotensin II [83, 84]. The previous studies have reported that the expression of mRNA in NOX1, NOX2, and NOX4 was increased in aortas from animals infused with Ang II [85, 86]. In addition, the expression of NOX2, NOX4, and NOX5 was found to be upregulated by Ang II in endothelial cells [87, 88], and knockout of NOX1, NOX2, and NOX4 in mice can reduce blood-pressure elevation induced by Ang II [86, 89, 90]. Taking together, these data suggest that activation of NOX1, NOX2, NOX4, and NOX5 play an important role in the development of Ang II-induced hypertension.

NOX2 is a major ROS source in the heart, and its activity increases after acute myocardial infarction (AMI). Compared with the normal human cardiomyocytes, the expression of NOX2 was higher in patients with AMI [91]. One study reported that the absence of NOX2 in Apoe-/- mice decreased ROS production, increased NO bioavailability, and reduced aortic atherosclerosis [92]. But Sirker et al. indicated that the overexpression of NOX2 in cardiomyocyte or endothelial cell made no difference to initial infarct size in mice with AMI at 4 weeks [93]. Vendrov et al. [94] suggest that expression of NOX4, but not NOX1 or NOX2, was correlated with increased mitochondrial oxidative stress, mitochondrial and cardiovascular dysfunction, and atherosclerosis in aged mice. The study indicated that NOX4 is a potential therapeutic target for aging-associated cardiovascular disease. The current study also demonstrated that NOX5 expression increased in patients with AMI, especially in infarctions > 12 hours [95]. In summary, the expression of NOX2, NOX4, and NOX5 plays a role in the development of coronary heart disease.

#### 3.2.2. NOX and Prostatic Diseases

Evidence has showed that the aberrant activation of NOX plays a critical role in prostate cancer growth and progression [96, 97]. A recent study showed that NOX expression is directly associated with prostate cancer in mice, and when NOX was inhibited, the expression of HIF-1*α* in the nucleus was significantly decreased as well as a reduction in the proliferation and colony formation of prostate cancer [98]. Some studies have shown that NOX1 and its transmembrane subunit P22^phox^ and NOX5 are overexpressed in prostate cancer [99–102], and downregulated NOX5 expression can inhibit cell proliferation and tumor growth and induce apoptosis of prostate cancer cells [103]. In addition, studies have reported that various isoforms of NOX are found in prostate cancer cell lines, including NOX4, NOX2, and NOX5, which are absent in normal prostate cell lines [104, 105]. An in vitro study has shown that the imbalance of redox homeostasis caused by elevated NOX4-derived ROS signal was the basis of fibroblast-to-myofibroblast differentiation in the diseased prostatic stroma, which indicates NOX4 inhibitors have potential clinical value in the prevention of BPH and prostate cancer [106]. Thus, NOX1, NOX2, NOX4, and NOX5 may be potential targets for therapeutic intervention in prostate cancer.

## 4. Conclusion

Evidence from clinical and animal studies demonstrates that CVD is associated with prostatic diseases, and oxidative stress may erect a bridge between these diseases; however, the exact mechanism of oxidative stress in CVD and prostatic diseases remains to be further elucidated. We provide a framework for future experimental and clinical studies on the role of known and yet to be discovered oxidative stress in CVD and prostatic diseases. The mechanisms and signalling processes by Ang II increased ROS production via NOX in these diseases is yet to be proved. Furthermore, NOX-derived ROS signals may be a common potential target in therapeutic intervention of CVD and prostatic diseases.

## Figures and Tables

**Table 1:** Epidemiological studies about the associations of CVD and prostatic diseases^a^.

Author (year)	Country	Study design	Disease diagnosis		Sample size	Age (year)	Main outcomes	Reference
			Cardiovascular diseases	Prostatic diseases				
Bourke J B, et al. 1966	UK	Case control	HP (SBP > 200 mmHg and DBP > 110 mmHg)	BPH (diagnosed histologically)	432	65-69	The incidence of HP in patients who were operated upon for BPH was significantly greater than control series.	[11]

Sugaya K, et al. 2003	Japan	Cohort study	HP (SBP ≥140 mmHg or DBP>90 mmHg)	BPH (digital rectal examination and ultrasonography)	42	NT group: 69 ± 8 HT group: 71 ± 11	HP may worsen LUTS.	[12]

Michel M C, et al. 2004	Germany	Case control	HP (DBP > 90 mmHg or with history of hypertension or receiving antihypertension medication)	BPH (diagnosed by urologist)	9857	Mean: 65.1	Patients with HP had more severe BPH symptoms and that more severe BPH symptoms are associated with a high HP.	[13]

Chen I H, et al. 2012	China	Case series	HP (the history of hypertension)	BPH (IPSS > 8 and PV > 18 cm^3^)	130	60.9 ± 10.8	The more cardiovascular risk factors in patients with BPH, the greater was the prostate vascular resistance.	[14]

Hwang E C, et al. 2015	South Korea	Case control	HP (SBP ≥ 140 mmHg or DBP ≥ 90 mmHg or with a previous diagnosis of hypertension and receiving medical treatment)	BPH (transurethral resection of the prostate)	295	69.5 ± 7.0	Men with HP were more likely to have greater LUTS and larger prostate volume.	[15]

Zeng XT, et al. 2018	China	Cross-sectional study	HP (NR)	BPH (NR)	350	NT group: 71.5 ± 7.4 HT group: 70.7 ± 7.3	HP had no significant association with prostate volume.	[16]

Navin S, et al. 2017	US	Cross-sectional study	HP (NR)	PCa (NR)	3200	51-76	Patients with PCa had a significantly higher prevalence of HP than the general population.	[17]

Dickerman B A, et al. 2018	Iceland	Cohort study	HP (SBP ≥ 140 mmHg or DBP ≥ 90 mmHg or taking anti-hypertensives)	PCa (morphologically verified)	9097	52.1 ± 8.4	This was a positive association between midlife hypertension and aggressive PCa.	[18]

Weisman K M, et al. 2000	US	Case control	CHD (included the history of coronary artery bypass graft, coronary angioplasty, and myocardial infarction)	BPH (prostate biopsy and transurethral resection of the prostate)	140	65-80	Patients without BPH had a lower frequency of CHD than those with BPH.	[19]

Neugut AI, et al. 1998	US	Case control	CHD (the history of myocardial infarction, coronary artery bypass graft, positive coronary angiogram, or positive exercise stress test)	PCa (diagnosed pathologically)	508	Case group: 69.6 ± 9.1 Control group: 68.1 ± 9.0	The individuals with CHD are at elevated risk for PCa.	[20]

Stamatiou KN, et al. 2007	Greece	Case serials	CHD (pathologic examination)	PCa (histological features)	116	55-98	There could be an association between CHD and PCa.	[21]
Thomas JA 2nd, et al. 2012	US	Clinical study	CHD (post history)	PCa (biopsy and PSA)	6729	50-75	CHD was significantly associated with PCa diagnosis.	[22]

Omalu BI, et al. 2013	US	Case serials	CHD (two forensic pathologists and a senior pathology resident)	PCa (two genitourinary pathologists for histologic)	37	65.8 (50-86)	There was no association between degree of CHD and PCa.	[23]

^a^HP: hypertension; BPH: benign prostatic hyperplasia; SBP: systolic blood pressure; DBP: diastolic blood pressure; NT: normotensive; HT: hypertensive; PCa: prostate cancer; IPSS: international prostate symptom score; LUTS: lower urinary tract symptoms; NR: not reported; PV: prostate volume; CHD: coronary heart disease.

**Table 2:** The summary of oxidative stress in CVD or prostatic diseases.

Author (year)	Study design	Study population	Age (year)	Markers assessed	Main results	Reference
Germanò G, et al. 2004	Cross-sectional study	40 persons with HP 40 healthy individuals	HP group: 51.6 ± 3 Healthy group: 54.4 ± 2	(i) O_2_^−^ measured by lucigenin chemiluminescence and hydroethidine cytofluorimetric	Patients with hypertension showed an enhanced formation of O_2_^−^ in platelets.	[37]

Guxens M, et al. 2009	Cross-sectional study	819 CHD patients with HP 311 CHD patients without HP	HP group: 67 ± 8 Control group: 66 ± 9	(i) Circulating ox-LDL measured by an enzyme-linked immunosorbent	There was a positive relationship between circulating ox-LDL and hypertension.	[38]

Pinzón-Díaz CE, et al. 2018	Clinical trial study	12 persons with HP 15 healthy individuals	26-50	(i) MDA by a spectrophotometer (ii) GSH concentration used the glutathione assay kit	Compared to healthy patients, the level of lipid peroxidation is higher 2.1 times in hypertensive patients.	[39]

Zhao H, et al. 2018	Clinical trial study	75 people with HP 75 healthy people	HP group: 40.41 ± 11.66 Control group: 40.08 ± 4.31	(i) Melatonin measured by metabolomic	Oxidative stress would cause disturbance in hypertensive patients and affect the metabolic pathway of pathogenesis.	[40]

Merendino RA, et al. 2003	Clinical study	22 patients with BPH 22 healthy subjects	BPH group: 65.8 (56-79) Control group: 62.1 (55-76)	(i) MDA measured a commercially kit	The results showed a higher level of MDA in BPH patients.	[44]

Camphausen K, et al. 2004	Cohort study	38 radiotherapy PCa cases 15 received placebo	NR	(i) Urinary 8-iso-prostaglandin PGF_2*α*_ and 15-keto-dihydro-PGF_2*α*_	The study showed that there was no statistically increase in 8-iso-PGF_2*α*_ or 15-keto-dihydro-PGF_2*α*_ in patients with PCa compared with normal control group.	[45]

Yilmaz MI, et al. 2004	Case-control study	50 patients with BPH 21 patients with PCa 50 healthy subjects	BPH group: 63.5 (43-84) PCa group: 66 (49-84) Control group: 66 (48-78)	(i) CuZn-SOD, and GPX measured by a UV–VIS recording spectrophotometer (ii) MDA	Compared with BPH and control groups, there is a higher MDA concentration with lower GPX and CuZn-SOD activities in PCa patients.	[46]

Srivastava DS, et al. 2005	Case-control study	55 patients with BPH 45 patients with PCa 25 healthy individuals	BPH group: 59.6 ± 8.4 PCa group: 61.9 ± 11.4 Control group: 60.5 ± 14.3	(i) GPX measured by kit (ii) MDA (iii) GST and GSH activities measured by spectrophotometry	Compared with control group, there is a higher level of MDA concentration and GST activity and lower levels of GSH concentration and GPX activity in BPH and PCa groups.	[47]

Aydin A, et al. 2006	Clinical study	36 patients with BPH 25 patients with PCa 24 healthy subjects	BPH group: 64.3 ± 7.9 PCa group: 67.5 ± 8.8 Control group: 65.0 ± 6.0	(i) The level of TBARS, SOD, GPX, CAT, Cu, and Zn	Compared with control group, the lipid peroxidation was increased with decreased SOD activity in BPH and PCa groups.	[48]

Surapaneni KM, et al. 2006	Case-control study	30 patients with PCa 30 healthy cases	NR	(i) MDA measured by spectrophotometry (ii) SOD measured by Misra and Fridovich (iii) GST and GSH activities measured by spectrophotometry	Compared with control group, there is a higher level of MDA and SOD and lower level of GSH in PCa patients.	[49]
Ozmen H, et al. 2006	Cross-sectional study	20 patients with PCa 21 healthy cases	PCa group: 72.45 ± 7.78 Control group: 66.33 ± 8.25	(i) MDA and vitamins measured by HPLC (ii) SE measured by a fluorimetric method (iii) Trace elements and Fe measured by atomic absorption spectrophotometry	The study showed that the administration of vitamins A, C, and E and SE and Zn may be beneficial in the prevention and treatment of human prostate cancer.	[50]

Lockett KL, et al. 2006	Case-control study	158 patients with PCa 128 healthy cases	PCa group: 65.3 ± 9.5 Control group: 64.4 ± 9.5	(i) DNA damage evaluated by alkaline comet assay	The study suggested that DNA damage may be associated with PCa risk.	[51]

Aryal M, et al. 2007	Case-control study	48 patients with BPH 46 healthy cases	BPH group: 67 ± 12 Control group: 63.4 ± 8	(i) MDA (ii) *α*-Tocopherol and ascorbate	Compared with control group, there is a higher level of plasma MDA and lower plasma alpha-Tocopherol and ascorbate level in patients with BPH.	[52]

Goswami K, et al. 2007	Case-control study	10 patients with BPH 10 patients with PCa 10 control subjects	BPH group: 65 ± 3 PCa group: 67 ± 4 Control group: 65 ± 7	(i) Lipid peroxide was estimated by spectrophotometry (ii) Protein carbonyls measured by modified Levine's	Compared with control group, there is a higher level of lipid peroxides and protein carbonyls in patients with BPH or PCa, and PCa patients are more prone to oxidative damage in compared with BPH patients.	[53]

Arsova-Sarafinovska Z, et al. 2009^a^	Case-control study	67 patients with BPH 73 patients with PCa 23 control subjects	BPH group: 64.3 ± 7.9 PCa group: 67.5 ± 8.8 Control group: 65.0 ± 6.0	(i) MDA (ii) CuZn-SOD, GPX, and CAT measured by a UV–VIS recording spectrophotometer (iii) NO2^−^/NO3^−^ (iv) 8-OHdG measured Highly Sensitive 8-OHdG Check ELISA Kit	Compared with BPH and control groups, there is a higher MDA and NO2^−^/NO3^−^ concentration with lower GPX and CuZn-SOD activities in PCa patients.	[54]

Arsova-Sarafinovska Z, et al. 2009^b^	Case-control study	100 patients with BPH 34 patients with PCa 15 control subjects	BPH group: 64.3 ± 7.9 PCa group: 67.5 ± 8.8 Control group: 65.0 ± 6.0	(i) MDA (ii) CuZn-SOD, GPX, CAT measured by a UV–VIS recording spectrophotometer (iii) NO2^−^/NO3^−^ (iv) 8-OHdG measured Highly Sensitive 8-OHdG Check ELISA Kit	Compared with BPH and control groups, there is a higher MDA and NO2^−^/NO3^−^ concentration with lower GPX and CuZn-SOD activities in PCa patients.	[54]

Pace G, et al. 2010	Case-control study	7 patients with BPH 11 patients with PCa 5 healthy subjects	BPH group: 65.14 ± 2.12 PCa group: 62.82 ± 1.74 Control group: 66.00 ± 3.51	(i) ox-LDL, peroxides, TEAC, and SOD measured in blood samples	The study confirmed a significant imbalance of redox status in patients with BPH and PCa and suggests that oxidative stress may be a determinant in the pathogenesis of these diseases.	[55]
Hoque A, et al. 2010	Nested case-control study	1808 PCa cases 1805 controls	Case group: 63.62 ± 5.54 Control group: 63.58 ± 5.55	(i) Serum protein carbonyl level measured by a noncompetitive ELISA	The study did not support that oxidative stress plays a role in PCa risk or its aggressiveness in serum protein carbonyl level.	[56]

Cimino S, et al. 2014	Case-control study	60 BPH patients 40 PCa patients	BPH group: 68 ± 6.4 PCa group: 67 ± 8.7	(i) The level of total thiol groups (TTG) and glutathione	A significant difference of TTG was observed in BPH and PCa patients, and the level of glutathione was lower in PCa patients.	[57]

HP: hypertension; CHD: coronary heart disease; O_2_^−^: superoxide anion; HIAE: high-intensity aerobic exercise; LIAE: low-intensity aerobic exercise; BFE: blood flow restriction; TBARS: thiobarbituric acid-reactive substances; SOD: the enzyme activities of superoxide dismutase; GPX: glutathione peroxidase; CAT: catalase; Cu: copper; Zn: zinc; NR: not reported; BPH: benign prostatic hyperplasia; PCa: prostate cancer; TEAC: total equivalent antioxidant capacity; ^a^the data from Macedonia; ^b^the data from Turkey; MDA: erythrocyte malondialdehyde; NO2^−^/NO3^−^: nitrite/nitrate; 8-OHdG: 8-hydroxy-2′-deoxyguanosine; GST: glutathione s-transferase.
